# The Sarcoma Immune Landscape: Emerging Challenges, Prognostic Significance and Prospective Impact for Immunotherapy Approaches

**DOI:** 10.3390/cancers13030363

**Published:** 2021-01-20

**Authors:** Anna Koumarianou, Jose Duran-Moreno

**Affiliations:** Hematology Oncology Unit, Fourth Department of Internal Medicine, Attikon University Hospital, Medical School, National and Kapodistrian University of Athens, 14561 Athens, Greece; duranmoreno.jose@gmail.com

**Keywords:** immunotherapy, T-cells, CAR-T cells, PD-L1, nivolumab, pembrolizumab, ipilimumab, chemotherapy, radiotherapy, targeted therapy

## Abstract

**Simple Summary:**

Sarcomas are a rare disease with high rates of recurrence and poor prognosis. Important discoveries about the biology of sarcomas have been done during the last decades, without a substantial improvement of systemic treatments. With the agnostic effectivity of immuno-oncological agents in different cancer indications, it is expected that sarcomas can also benefit from these treatments. This article gathers the available data on the specific immune tumor microenvironment of sarcoma and the immunotherapeutic strategies currently under investigation.

**Abstract:**

Despite significant advances in multidisciplinary treatment strategies including surgery, radiotherapy, targeted therapy and chemotherapy there are yet no substantial improvements in the clinical benefit of patients with sarcomas. Current understanding of the underlying cellular and molecular pathways which govern the dynamic interactions between the tumor stroma, tumor cells and immune infiltrates in sarcoma tissues, led to the clinical development of new therapeutic options based on immunotherapies. Moreover, progress of the treatment of sarcomas also depends on the identification of biomarkers with prognostic and predictive values for selecting patients most likely to benefit from these new therapeutic treatments and also serving as potent therapeutic targets. Novel combinations with radiotherapy, chemotherapy, targeted therapy, vaccines, CAR-T cells and treatments targeting other immune components of the tumor microenvironment are underway aiming to bypass known resistance mechanisms. This review focuses on the role of tumor microenvironment in sarcoma, prognosis and response to novel immunotherapies.

## 1. Introduction

Sarcomas are very heterogenous tumors, with more than 100 histologic subtypes characterized by the evolving recognition of distinct morphological and genetic features [1,2]. Based on a recent analysis of the Surveillance, Epidemiology and End Results cancer database, the incidence of sarcomas has been reported to increase from 6.8 cases/100.000 individuals in 2002 to 7.7 in 2014 whereas the median 5-year survival of patients with metastatic disease has remained at 30% [3]. Selected epidemiological data on the incidence and survival of sarcoma subtypes are shown in Table 1. It is thus very important to investigate novel biomarkers acting as potent therapeutic targets so to apply new treatments with improved clinical efficacy. In this frame, immunotherapies may provide new treatment options for sarcomas. However, due to the vast molecular heterogeneity of sarcomas clinical efficacy may widely vary among patients and thus the identification of patients most likely to respond to immunotherapies is of great importance. Predictive biomarkers for immune checkpoint blockade have been proposed and their validation in clinical trials will be most useful to guide selection of patients to benefit from immunotherapies and in this way to modify the landscape of this disease. To this end, the level of tumor-infiltrating lymphocytes (TILs) and programmed cell death-ligand 1 (PD-L1) expression have been correlated with prognosis in both soft tissue sarcomas (STS) and bone sarcomas (BS) [4]. Tumor-associated antigens (TAA) including cancer testis (CT) antigens have been reported to induce immune responses in STS patients thus providing an important therapeutic target for this type of cancer [5,6]. In addition, the predictive and prognostic role of tumor mutational burden (TMB) in sarcoma is under intensive investigation. 

This review presents the rationale, design and clinical efficacy of newest therapeutic approaches, incorporating immunotherapeutic modalities for sarcomas. 

## 2. The Immune Tumor Microenvironment in Sarcomas

The immune system has the potential to identify and destroy nascent tumor cells in a process named cancer immunosurveillance [12]. The process is initiated by innate immunity involving cells of the immune system such as macrophages, dendritic cells, myeloid-derived suppressor cells and natural killers [13]. T-cell priming and activation of effector T-cells against tumor cells constitute an important part of the adaptive immunity [5]. However, the interplay between innate and adaptive immune system can also promote tumor progression [5]. Together, the dual host-protective and tumor-promoting actions of immunity are referred to as cancer immunoediting. According to the immunoediting theory the tumor microenvironment (TME) represents the prominent site where tumor evolution is taking place based on continuous and dynamic interactions mainly between tumor cells and elements of the immune system [6]. The relative balance of effector and memory immune cells, on one hand, and immunosuppressive populations in the TME, on the other, determines the fate of the tumor [14]. In sarcomas, the TME is highly immunosuppressive with high densities of hypoxia-inducible factor 1 α (HIF1α), macrophages, neutrophils and decreased T-cell levels [15]. A previous study has also implicated tumor-associated macrophages (TAMs) in the establishment of an immune-hostile TME promoting the growth, angiogenesis and metastasis of sarcomas posing an obstacle for the development of an effective antitumor adaptive immunity [16]. The activation of the STAT3 pathway, besides promoting the immunosuppressive effect of myeloid-derived suppressor cells, has an antiapoptotic effect and confers insensitivity to chemotherapy mediated by the secretion of IL-22 by T-cells [17,18]. An immunosuppressive TME can also be characterized by programmed cell death protein-1 (PD-1) and PD-L1 expression which dampen adaptive antitumor immune responses. In a recent systematic meta-analysis of 14 studies including 868 patients with STS and BS, the expression of PD-L1 was positively correlated with the infiltration of PD-1 positive T-lymphocytes and significantly correlated with metastasis, mortality risk, and poorer overall survival in patients with BS [19]. 

The relatively non-immunogenic phenotype attributed to sarcomas could be due to the low-intermediate TMB displayed by some STS subtypes as compared to the other cancer types [20]. On the other hand, non-synonymous somatic mutations are coding for neoantigens, exclusively expressed by cancer cells, which could induce potent immune responses, because the quality of the T-cell repertoire recognizing these antigens in the context of particular major histocompatibility complex (MHC) alleles is not affected by central T-cell tolerance [21]. Some subtypes such as undifferentiated pleomorphic sarcoma (UPS) have a higher number of non-synonymous mutations and greater TMB opening a window of opportunity for immunotherapeutic approaches such as vaccines and anti-PD-1/PD-L1 therapies [21].

## 3. Tumor-Associated Antigens

TAA are cellular-membrane bound proteins characterized by restricted normal tissue expression profile and heterogeneous expression by cancer cells. Stimulation of a tumor-specific immune response after the recognition of TAA is the rationale of therapeutic vaccines. To date, several potential TAA have been identified in sarcomas. CT antigens are molecules exclusively expressed by germ cells and so the immune system cells recognize them as foreign antigens [22]. There are different CT antigens and their overexpression seems to vary from one sarcoma subtype to another. Such examples are NY-ESO-1 and MAGE in high-grade STS [23]. Gangliosides are cell-surface molecules composed of a glycosphingolipid and at least one sialic acid residue. Among the members of this vast protein family, disialogangliosides GD2 and GD3 are expressed in neural and mesenchymal stem cells but, postnatally, their expression is restricted to peripheral neurons, central nervous system and sarcomas [24]. In addition, around a 20% of sarcomas are associated to chromosomal translocations, which create oncogenic fusion genes encoding for fusion proteins [25]. These unique properties enable the utilization of TAA as targets to develop tumor specific immune responses. 

## 4. Biomarkers for Immunotherapy in Sarcoma

### 4.1. Intratumoral Immune Infiltrates and PD-L1

Tumor immune cell infiltrates, such as effector CD8+ T-cells, are essential in hindering cancer progression and may complement the classification systems of cancer, as in the case of breast cancer and melanoma [26,27]. Immunological biomarkers are preferentially explored for immunotherapies as a result of the immune contexture of the TME and our comprehensive knowledge about the pathways which are targeted by immunotherapy and in particular by immune checkpoint inhibitors (ICI). IFNγ produced by activated cells of the innate and adaptive immunity induces the expression of PD-L1 on the surface of tumor cells and myeloid cells such as macrophages and myeloid-derived suppressor cells [16]. PD-L1 binds on its natural ligand PD-1 which is expressed by the activated CD8 + cytotoxic T-lymphocytes (CTLs) and de-phosphorylates substrates downstream the nucleus, resulting in reduced proliferation and cytokine production [28]. Administration of anti-PD-1 and/or anti-PD-L1 monoclonal antibodies (MAbs) unblocks immune inhibition via PD-1/PD-L1 bridging and enhances tumor cell killing by the CD8 + CTLs [28]. As thoroughly addressed in this review, multiple clinical trials have addressed the therapeutic efficacy of ICI, including ipilimumab, nivolumab, pembrolizumab and atezolizumab, in sarcoma patients [29,30,31,32,33,34]. Although producing remarkable clinical responses, ICI are effective in a rather low percentage of cancer patients and therefore the identification of biomarkers for selecting patients most likely to respond to ICI is of paramount importance.

Although the role of intratumoral CD8 + T-cell densities in sarcomas as prognosticators has not yet been firmly established, levels of PD-1 + CD8 + (TILs and intratumoral expression of PD-L1 in sarcoma subtypes have been correlated with prognosis [35,36], suggesting that PD-1/PD-L1 interaction could regulate T-cell-mediated control of tumor growth and pointing to the use of ICI for sarcomas. To this point, it should be mentioned that PD-L1 expression might not always be a reliable biomarker for predicting responses to ICI. This was better shown in a large phase III study with advanced-stage melanoma patients but also in a meta-analysis of anti-PD1/PD-L1 clinical trials across different malignancies indicating that clinical responses to immunotherapies could be detected also among patients with PD-L1 negative tumors [37,38]. PD-L1 can be expressed in tumor cells as a result of genetic modifications including PTEN loss, EGFR mutations, MYC overexpression, mutations in the PI3K/AKT signaling pathway and PDJ amplification [39]. Interestingly, in a recent report it was shown that not PD-L1 per se, but rather the composition of the TME in which PD-L1 is induced, determines tumor recurrence [40]. In the same study it was found that PD-L1 + tumor cells generated in the context of activated TAMs were resistant to chemotherapeutic regimens and therapy with ICI, whereas PD-L1 + tumor cells in the context of activated T-cells producing IFNγ, were sensitive to ICI. The role of tertiary lymphoid structures and a proposed immune classification system in patients with sarcoma was underscored in a recent study [41]. Based on the immune cell composition of the TME and the expression of gene signatures related to the functional orientation of the immune TME and to immune checkpoints, sarcomas were classified as A “immune desert”, B “immune-low profile”, C “vascularized”, D “immune-high profile” and E “immune and CTLS high”. Patients in class E, characterized by the presence of tertiary lymphoid structures containing T cells, follicular dendritic cells and B cells, demonstrated improved survival and high response rate to pembrolizumab [41].

### 4.2. Tumor Mutational Burden (TMB)

High TMB is largely being considered as a response biomarker to modern immunotherapeutics across different malignancies [42]. In recent years, high-throughput sequencing technologies have made it possible to detect somatic mutations in tumors and to identify TMB profiles. The numbers are still being defined, but, in general, 20 mutations per Mb (m/Mb) are considered high [43]. Accumulated evidence indicates large heterogeneity of TMB among sarcoma subtypes ranging from very low (0.15 m/MB) to high (29 m/MB) [43,44,45,46]. Most of the large cohort studies indicate that undifferentiated and high-grade sarcoma subtypes are most likely to exhibit TMB high values [43,46]. A recent analysis of 133 sarcoma samples, mostly low grade, identified only two cases with high TMB; one UPS and one high-grade LMS [47]. In another study, characterization of TMB in a large sarcoma cohort including different subtypes, identified a median TMB of 1.7 in most patient and TMB high in only 2% of patients [48]. In a homogeneous study including tissue samples from 26 cardiac sarcomas, whole exome sequencing and NGS analysis identified high TMB in 92.3% of patients [45]. In another study investigating the TMB values in 16 patients with UPS who underwent complete local resection twice due to relapse identified a statistical increase of the TMB after recurrence (4.6 vs. 7.5 m/MB) [49]. A recent study identified a subgroup of synovial sarcomas with high TMB and suggested that this might explain the 10% response rate to ICI observed in clinical trials including patients with synovial sarcoma [50].

The predictive and prognostic role of TMB in sarcoma has not been fully elucidated. A case series reported results on heavily pretreated with chemotherapy angiosarcoma patients with one patient demonstrating CR on anti-CTLA-4 therapy with low TMB (0.09 mutations/mb) and 2 patients achieving partial responses on pembrolizumab and nivolumab/ipilimumab with high TMB (12 and 15 mutations/mb) [51]. A retrospective analysis of TMB as predictive marker of response to anti-PD1 therapy identified 1 patient (out of 3) with UPS with intermediate TMB who responded to therapy [42]. Another recent report indicated that patients with TMB high and elevated effector immune cells infiltrate exhibited the highest survival [52]. 

Further proof-of concept studies are required to determine which specific biomarkers including TMB are clinically meaningful to predict the efficacy of anti-PD1 blockade in sarcomas.

## 5. Immunotherapeutic Strategies

### 5.1. Monoclonal Antibody Therapy

Several MAbs have been introduced in the therapeutic armamentarium of tumors aiming to manipulate critical signaling pathways that sustain their malignant phenotype and metastatic potential such as olaratumab and denosumab [53,54]. These MAbs belong to the passive immunotherapy approach and differ to ICI that are able to elicit and maintain specific immune responses against cancer cells and thus represent an active immunotherapy modality [55,56].

Different MAbs have been tested for the treatment of sarcomas. The antiangiogenic agent olaratumab, a recombinant IgG1 MAb targeting the subunit alpha of platelet-derived growth factor receptor that has been tested in a phase III study in STS combined with doxorubicin vs. doxorubicin alone, did not improved overall survival over doxorubicin alone resulting in its removal from the market [54]. 

Denosumab is a MAb targeting the receptor activator of nuclear factor kappa-B ligand. Patients with giant-cell tumors of the bone showed clinical responses to denosumab that has now become the standard therapy for this disease [53].

Disialogangliosides (GD)-2 and -3 have become an attractive target in sarcomas, since their expression in tumors of neuroectodermal origin and in different histological subtypes of sarcoma was established [57]. The efficacy of MAbs targeting GD2 in neuroblastoma has led to the approval of dinutuximab as consolidation treatment for patients with neuroblastoma [58] and incited the research of newer, bispecific antibodies. A recent preclinical study on GD2 and HER2 overexpressing osteosarcoma cell lines and xenograft mouse models indicated that the administration of anti-GD2xanti-CD3 and anti-HER2xanti-CD3 bispecific antibodies was associated with substantial T-cell recruitment in the tumor and potent antitumor activity [59]. 

The overexpression of a different targetable receptor, the insulin-growth factor-1 receptor (IGF-1R), has been described in tumors associated to the pubertal growth spurt [60]. Its overexpression in Ewing sarcoma has led to an extensive research of the IGF-1R-inhibitor ganitumab, which has shown a clinical benefit rate of 36.8% in monotherapy in a phase II clinical trial [61]. Results of ongoing studies with ganitumab in combination with palbociclib (NCT04129151) and chemotherapy (NCT02306161) are awaited.

### 5.2. Immune Checkpoint Inhibition

Immune checkpoint inhibitors (ICI) are robustly modulating cellular antitumor immunologic responses and therefore are considered as the mainstay of current and future therapeutic modalities for cancer. Nevertheless, the number of patients responding to ICI is lower than anticipated and among responders there is a substantial number of patients who develop immune resistance [39]. Consequently, it is conceivable that we must consider additional steps in the immune pathways underlying the immunomodulatory effects caused by ICI to understand if and to what extent combinatorial or sequential strategies will be most capable for improving immune efficacy and blocking or reversing immune resistance mechanisms. To this end, PD-L1 and TMB have emerged over the past few years as potential biomarkers for selecting patients most likely to benefit from ICI [62]. 

A study testing the anti-CTLA-4 ICI ipilimumab, that in other cancers was shown to induce strong immune responses, has shown low or no response in sarcoma patients. This study was one of the first to indicate primary resistance to immunotherapy [63]. Subsequent clinical data from the Alliance phase II trial, including metastatic sarcoma patients, treated with the anti-PD1 ICI nivolumab alone with or in combination with ipilimumab, showed a response rate of 5% and 16% respectively and these were independent of PD-L1 expression [29]. Activity was seen in patients with UPS, leiomyosarcoma, myxofibrosarcoma, angiosarcoma, and alveolar soft-part sarcoma [29].

A single arm, phase II clinical study (SARC028) with pembrolizumab in heavily pretreated patients with STS and bone sarcoma did not meet the primary endpoint of overall response [20]. However, pembrolizumab showed encouraging activity in patients with undifferentiated pleomorphic sarcoma or dedifferentiated liposarcoma [20].

Several cell-intrinsic and -extrinsic factors may contribute to these forms of immune checkpoint blockade resistance. The percentage of sarcomas expressing PD-L1 is moderate with a wide range of variation among the tumor cells expressing PD-L1 which poses a serious obstacle for the use of PD-L1 as a prognostic biomarker [64]. High TMB has been suggested to associate with microsatellite instability and increased immunogenicity which confer substantial clinical responses to anti-PD1 treatment [62]. Although there is a lot of controversy with regard to the cutoff defining high TMB, in general lines increased TMB has been shown to correlate with favorable clinical outcomes across multiple cancer types also including sarcomas [65].

Generally, limited evidence exists on the activity of ICI in sarcoma with low clinical activity of anti-PD1 alone in most sarcoma subtypes suggesting that blocking the PD1 negative signaling alone is not sufficient to adequately reactivate the exhausted endogenous antitumor effector T cells in these patients [35]. To this end one should consider that sarcomas are quite heterogenous, with many subtypes, which makes conceivable that and the TME is highly complicated making extremely uncertain any prediction for the outcome of immunotherapies. This may satisfactorily justify why PD-1 blockade monotherapy cannot achieve the desired efficacy based on the so far available data. Through detailed analyses on the TME of sarcomas, it may be possible to identify patients who are responsive to PD-1 therapeutic treatment by examination of expression of comprehensive bio-signatures also including PD-L1 expression, TMB, and MSI.

### 5.3. Strategies to Improve the Immunogenicity of Sarcomas

The greater understanding of the process of T-cell activation against sarcoma and sarcoma-associated antigens have paved the way for the development of newer strategies with strong potential for clinical application in the immediate future. It has been previously suggested the possibility to increase clinical efficacy for sarcoma patients by combining ICI with other treatment modalities, such as chemotherapy or radiation therapy, and targeted therapies [66]. The rationale supporting the use of such modalities is their capacity to decrease tumor burden and induce immunogenic tumor cell death with the release of DC-maturation factors along with TAA all of which enhance the potency of ICI to re-activate endogenous antitumor immune responses with clinical benefits for the patients. While surgery is a common treatment for many solid cancers, there is evidence that it impacts the rate of systemic tumor cell dissemination into blood vasculature and immune suppression [67]. To combat these negative events, ICIs are being explored as neoadjuvant therapies in combination with other downsizing treatment modalities such as radiotherapy and chemotherapy [68] (Table 2).

#### 5.3.1. Radiotherapy

Radiotherapy can sensitize tumor cells to ICIs by inducing immunogenic cell death, as a result of phagocytosis of tumor cells, processing of tumor antigens, and priming of CD8+ T cells [69]. In addition, radiotherapy has been observed to have a systemic effect on the reduction of tumors outside the initial treatment field, termed an abscopal effect (the shrinkage of untreated sites occurred concurrently with shrinkage of tumors within the field of RT, indicating a distant, possibly immunological effect) [70]. Table 2 lists relevant studies of radiation therapy combined with ICI.

#### 5.3.2. Chemotherapy

Chemotherapy has the potential to induce antigen release and immunogenic cell death [71]. The optimal timing of immune checkpoint inhibitor administration is under investigation (Table 2). One of the first studies investigating pembrolizumab in combination with metronomic chemotherapy in sarcomas was associated with limited activity in selected STS and GIST [34]. This was explained by an immunosuppressive tumor microenvironment resulting from macrophage infiltration and IDO1 pathway activation [51]. A recent study evaluated the safety and efficacy of doxorubicin in combination with pembrolizumab in patients with advanced, anthracycline-naive sarcomas [33]. The study indicated a 19% response rate and a median OS of 27.6 months (95%CI, 18.7-not reached). A correlative study indicated that TILS were present in 21% of evaluable tumors and associated with inferior PFS (log-rank *p* = 0.03), regardless of age, gender and previous treatment [33].

Another study evaluated the clinical impact of intra-tumoral infiltration of PD1-positive lymphocytes and PD-L1 expression in STS and found that both were associated with shorter event-free survival and poorer overall survival [35]. Ongoing studies of immunotherapy with chemotherapy are detailed in Table 2.

#### 5.3.3. Targeted Therapy

Sarcomas have diverse molecular characteristics and targeted therapies for these rare cancers are being investigated [72]. Targeted therapies have the potential to produce significant tumor response by disrupting molecular pathways that drive oncogenesis, thus providing more efficient and well-tolerated treatment [9]. However, to date, targeted therapy of sarcomas has only been partially effective [8]. Table 1 describes selected molecular targets and established or explored targeted therapies. 

The use of small-molecule therapies have been recently emerged as suitable candidates for combination therapies with ICI in overcoming some major ICI resistance constraints [73]. Targeted therapies with small molecules exhibit a number of advantages such as distinct toxicity profiles compared to ICI, shorter half-lives that reduce the chance of lasting systemic side-effects and target intracellular proteins making them an ideal candidate for partnering with ICI [73]. There are robust data pointing out that the inhibition of known, oncogenic intracellular pathways modifies the immune TME in malignancies such as non-small cell lung cancer and melanoma [74]. Although this relation has also been addressed in pathways considered hyperactivated in various subtypes of sarcoma, such as NOTCH and WNT/beta-catenin, most of the research available to date is related to the immunologic effects of MAPK-pathway inhibition [74,75,76,77]. Targeted treatment of EGFR-mutated non-small cell lung cancer may trigger an increase in TMB and an increase in tumor PDL1 expression [78]. In BRAF-mutated melanoma, the inhibition of BRAF and MEK proteins enhances the expression of melanocyte-differentiation antigens, the upregulation of proinflammatory cytokines, the downregulation of anti-inflammatory cytokines, the expression of PDL1 and the infiltration of the tumor by CD8^+^ T-cells [75]. These mechanisms set the basis of clinical studies with combination of targeted therapy and ICI [79]. These mechanisms are likely to be present also in sarcoma, but confirmatory data are needed. To date, two studies of targeted therapy in association with ICI in sarcomas are under way (Table 2).

Combining anti-angiogenesis drugs that reduce the growth of blood vessels and immune checkpoint inhibitors that promote the activation of cancer-killing immune cells offers a promising new therapeutic regimen for patients with cancer [80]. Several preclinical and clinical studies have indicated the existence of a molecular crosstalk between blood vessels and immune cells in the tumor microenvironment [80]. More specifically, (i) the disorganized network of tumor vessels hinder CD8 + T cell trafficking into the TME; (ii) VEGF interferes with the maturation of dendritic cells and thus suppresses T-cell priming; and (iii) protumoral M2-like macrophages as well as type-2, T-helper cells and regulatory T-cells (Tregs) secrete pro-angiogenic factors that accelerate uncontrolled angiogenesis and promote vascular immaturity thus creating a vicious circle [80].

Recent phase II and phase III clinical trials provided evidence of the clinical effectiveness of combining immunotherapy with antiangiogenic therapy leading to the regulatory approvals of pembrolizumab along with axitinib or lenvatinib for patients with kidney and endometrial cancer respectively [81,82]. In the field of sarcoma, the modification of immune TME with antiangiogenics has also gained attention. A single arm, phase II trial aimed to assess the activity of the VEGF receptor tyrosine-kinase inhibitor axitinib and pembrolizumab in 36 patients with pretreated sarcoma [83]. The study reported preliminary activity in patients with advanced sarcomas, particularly for patients with alveolar soft-part sarcoma paving the way for further investigation in randomized controlled trials [83]. A similar single-arm, phase Ib/II trial enrolled adult patients with selected subtypes of sarcoma investigated sunitinib and nivolumab [32]. The toxicity was mild, the overall response rate (ORR) was 21% and the PFS at 6 months was 48% [32]. 

### 5.4. Cellular Immunotherapy

#### 5.4.1. CAR T-Cells

Cellular immunotherapy is at this moment a “hot” field of research in the field of solid tumors, though at its infancy. Chimeric antigen receptor (CAR) modified T cells constitute an emerging, cellular adoptive immunotherapeutic modality with the advantages of bypassing restrictions of MHC presentation [84]. The impressive clinical efficacy achieved via CAR-T cell immunotherapy in hematologic tumors resulted to the application of this type of cellular therapeutic strategy in solid tumors also including sarcomas [84]. Compared to lymphomas and leukemias in solid malignancies CAR-T cell immunotherapy yielded only poor clinical results. While CAR-T cells targeting various sarcoma-associated antigens have been quite efficient in rejecting sarcoma cell lines in experimental tumor models, verification within clinical trials has been largely unsuccessful [84]. There are several reasons to explain this failure including (i) limited capacity of CAR-T cells to penetrate the TME resulting to inadequate infiltration; (ii) suppressor factors and cells within the tumor stroma including suppressor enzymes and tumor-associated macrophages, MDSC, Tregs or Bregs; and (iii) exhaustion of CAR- T cells via chronic exposure to TAA resulting to their functional dysfunction. Therefore, it is imperative to improve the efficacy of this therapeutic modality in a multifaceted manner by identifying optimal tumor-associated antigens, infusion protocols, lymphodepletion regimens, and combination protocols with the aim to improve clinical efficacy while minimizing toxicities. A recently completed clinical trial (NCT01343043) of genetically engineered NY-ESO-1 specific NY-ESO-1^c259^T-cells in HLA-A2+ patients with synovial sarcoma (NY-ESO-1+) indicated trafficking of these T-cells in the TME and maintained cytotoxicity in a subset of patients [85]. Table 3 presents ongoing CAR-T cell clinical trials aiming to explore the targeting of surface antigens of sarcoma cells with modified memory T cells such as 4SCAR-IgT cells (NCT03356782), CCT301-59 T cells (NCT03960060), C7R-GD2 T cells (NCT03635632) or NY-ESO-1 T cells (NCT03638206). A more complex study compares 2 CAR T cell strategies, one approach includes T cells directed at EGFR and the second T cells directed against EGFR and CD19, a marker on the surface of B lymphocytes. The latter is based on the hypothesis that CD19 + B cells serve in their normal role as antigen presenting cells to T cells and are expected to promote the expansion and persistence of the CAR T cells with specificity against EGFR (NCT03618381). Another study is exploring the effect of HER2 (Human Epidermal Growth Factor Receptor 2) CAR T cells that in addition contains CD28, which stimulates T cells and make them last longer (NCT00902044). The role of lymphodepleting chemotherapy (cyclophosphamide or fludarabine) is being investigated in parallel in a subpopulation of patients in most of these studies.

#### 5.4.2. Implications for Cellular Therapies Other than CAR T

In the hopes of extending this effort, other immune cell-based therapies are in current development. These include non-CAR/non-TCR gene modified cell therapies for cancer, such as the ex vivo activation of tumor infiltrating lymphocytes, and immunotherapies generated from less frequently studied cell types such as dendritic and natural killer (NK) cells [86]. The later type of cell is currently being developed as a potential cell therapy in the field of sarcoma. The great interest around NK cells is driven mainly by their capability of killing target cells autonomously and serve as the main effector cells toward cancer in innate immunity. A recent study aiming at the characterization of freshly dissociated sarcomas revealed a general decrease in tumor-infiltrating NK cells compared to the periphery, suggesting a defect in the endogenous NK cell response [87]. In the same study the genetic modification of NK cells to overexpress the activating receptors, DNAM-1 or NKG2D, elicited a dynamic increase in NK cell degranulation against all sarcoma explants in vitro [59]. In another preclinical model of pediatric sarcoma, the combination of oncolytic measles vaccine virotherapeutics together with IL-2 preactivated human NK cells resulted in an increased release of granzymes, perforin, and granulysin from NK cells [88]. An animal sarcoma model studied the effect of radiotherapy (RT) on ex vivo IL-2 preactivated and reinfused NK cells [89]. The study indicated that the NK cell homing and cytotoxicity were increased following RT indicating possible abscopal effects.

An additional approach to expand and activate NK cells has been described with histone-deacetylase inhibitors (HDACi) [90]. HDACi are epigenetic modifying agents that can inhibit sarcoma growth by inducing tumor cell apoptosis, cell cycle arrest, impairing tumor invasion and preventing metastasis. Besides the upregulation of tumor suppressor genes and the downregulation of oncogenes, the HDACi can modulate the immune response and enhance the cytotoxicity of NK cells in sarcoma cell lines by increasing the expression of NKG2D [90,91]. Based on this rationale, exploration of combinations of HDACi and immune cell-based therapies is warranted. The combination of the HDACi entinostat and NK cell infusion in nude mice with osteosarcoma lung metastases failed to demonstrate an enhanced anti-tumor activity of NK cells [92]. However, further research is needed to elucidate how HDACi can modulate the complexity of the anti-tumor immune response.

#### 5.4.3. Other Therapies Modifying Tumor Microenvironment

After recruitment, TAMs are “polarized” by different signals in the TME to classic-activated (M1) or alternative-activated macrophages (M2), exerting pro-inflammatory and anti-inflammatory function, respectively [93]. The best-known drug capable of stimulating the antitumor activity of macrophages is mifamurtide, a fully-synthetic derivative of a component of cell walls from Mycobacterium species. By binding the nucleotide-binding oligomerization domain-containing protein 2 (NOD2), mifamurtide triggers the production of proinflammatory cytokines [94]. The addition of mifamurtide to ifosfamide as adjuvant treatment for patients with localized osteosarcoma was investigated in a 2 double dagger 2 factorial phase III clinical trial of 677 patients. The addition of both mifamurtide and ifosfamide resulted in a 3-year event-free survival rate of 78% compared to the 71% achieved with standard treatment [95]. More recent approaches for repolarizing TAMs are focused on the colony-stimulating factor-1 receptor (CSF-1R), which has been pointed as responsible of M2-polarization [96]. The CSF-1R inhibitor pexidartinib was tested in a phase I clinical trial of 11 patients where a malignant fibrous histiocytoma, a sacral chordoma and a tenosynovial giant cell tumor (TGCT) were included [97]. The patient with TGCT achieved a long-lasting response, an expected effect due to the recurrent translocation t (1; 2) (p11; q35-36) leading to the fusion of CSF1 to COL6A3 that characterized this tumor [98]. With this rationale, pexidartinib and the anti-CSF-1R MAb emactuzumab have been investigated on TGCT in different phase I studies [99,100]. These results led to a phase III clinical trial where 120 patients were randomized to receive pexidartinib or placebo. The inhibition of CSF-1R in this trial achieved an overall response of 39% (95% CI 28–52), including 9 complete responses at week 25, as well as improvement in functional outcomes [101].

Apatinib is a vascular endothelial growth factor receptor 2 inhibitor shown to have immunomodulatory effect in sarcoma, as it reduces PD-L1 expression in osteosarcoma cells [102]. Apatinib was tested in a phase II trial including 59 patients with metastatic sarcoma (34.4% BS, 65.6% STS) showing clinical benefit (SD+PR) of 85.44%, median PFS of 7.93 months and median OS of 17.27 months [103].

A recent phase II clinical trial, investigated the combination of talimogene laherparepvec (T-VEC), that has been previously shown to increase tumor-specific immune activation via augmenting antigen presentation and T-cell priming, and pembrolizumab [30]. The study included 20 pretreated patients with advanced sarcoma of different histologic subtypes and reported a 35% objective response rate and a median duration of response of 56 weeks with a manageable safety profile [30].

### 5.5. Combining Vaccination with Other Therapeutic Modalities

Therapeutic cancer vaccines aim at re-activating preexisting antitumor immunity which is mediated by memory T cells and/or generating novel antitumor responses among the pool of naïve T cells. To be successful vaccines should specifically target immunogenic peptides expressed by the tumor in the context of MHC class I and class II molecules. Sarcomas are considered to be generally poor immunogenic and therefore there is a limited application of vaccines which is usually combined with other therapeutic modalities. In a phase I trial, a vaccine consisting of autologous mature DCs pulsed with peptide mixtures with overlapping sequences from NY-ESO-1, MAGE-A1, and MAGE-A3 cancer testis antigens is designed for the treatment of relapsed high-risk neuroblastoma, Ewing’s sarcoma, osteogenic sarcoma, rhabdomyosarcoma or synovial sarcoma post chemotherapy with decitabine (NCT01241162). Safety and antitumor immune responses are the primary and secondary endpoint from this trial, respectively. In another phase I study, a vaccine consisting of mature DCs pulsed with whole tumor cell lysates is combined with gemcitabine for the therapeutic treatment of patients with STS and osteosarcoma (NCT01803152) with safety as primary endpoint and reduction in the numbers of MDSCs and generation of immunological responses, as secondary endpoints. Autologous DCs were intratumorally injected as a cellular vaccine in a phase I study in patients with STS combined with fractionated external beam radiation. This combined treatment resulted in the generation of significant antitumor responses but clinical efficacy has not been tested [104]. In another study, tumor cell lines were used as therapeutic vaccines which were established from cancerous tissue from patients with metastatic cancer also including STS. This type of cellular vaccine was administered in combination with either IFNγ or GM-CSF as adjuvants. Both schedules were well tolerated and induced immunological response in vivo measured as DTH which were correlated with clinical efficacy [105]. Preexisting IgG immunity against tumor antigens was used as a criterion for selecting immunogenic tumor peptides to be used as personalized vaccines in patients with refractory sarcoma. Patients experienced no side effects and exhibited a reduction of tumor load in metastatic sites, with disease stabilization taking place in 30% of those [106].

## 6. Conclusions and Perspectives

Sarcomas represent a rare and heterogeneous group of cancers that even when treated radically and early they may progress to display intrinsic resistance to current systemic regimens. Single agent ICI studies have shown to be largely ineffective with only few patients responding and these are mainly with aggressive subtypes of sarcomas such as UPS. Several immunological studies have corroborated the poor immunogenicity and also the unfavorable immune-modulating nature of this malignancy. Novel strategies involving combination therapies with ICI and chemotherapy, radiotherapy or targeted therapies attempt to increase the immunogenic cell death. More advanced approaches manipulating the immune system such as polarization of macrophages, dendritic cells, NK cells and CAR T cells are also expected to shed more light on this heterogeneous disease. 

The presence of a large mutational heterogeneity has been recently reported among patients with the same type of sarcoma. This fact highlights the importance of identifying molecular biosignatures that can be used for the design of proper therapeutic approaches. This complexity of sarcoma molecular heterogeneity combined with the network of dynamic interactions of a variety of cell types within the TME underlines the requirement for precision therapies which likely will induce clinical benefits in sarcoma patients. For instance, cancer genomics could provide the platform for successful personalized oncology in sarcoma, introducing combinatorial treatments, targeting various components within the TME. 

In the near future, personalized therapy in oncology may be achieved through the customization of the right combinatorial treatment based on identified tumoral and personal features. Several translational and clinical studies are ongoing to identify and validate potential predictive biomarkers. In particular, in the case of sarcomas it is mandatory to explore therapeutic modalities which will integrate these biomarkers to overcome not only the obstacle of low immunogenicity of sarcomas but also therapeutic resistance.

## Figures and Tables

**Table 1:** Selected clinical and molecular characteristics of sarcomas with potential targeted therapies [3,7,8,9,10,11].

Histology	% of Patients	5-y CSS (%)	Molecular Targets	Targeted Therapy
Sarcoma, NOS	14.8	55.2	TK, ALK, NTRK	sunitinib, cediranib, anlotinib, tivatinib pazopanib, crizotinib, entrectinib
Leiomyosarcoma	14.6	60.5	genomic instability	pazopanib
Liposarcoma	11.3	82.8	CDK4, MDM2, p53, TK	Palbociclib, RG7112, selinexor, sitravatinib
Gastrointestinal stroma tumors	10.8	80.4	KIT, PDGFRA	Imatinib mesylate, sunitinib, sorafenib
Malignant Fibrous Histiocytoma	7.3	77.0	ALK, SQSTM1-ALK, VCL-ALK	crizotinib
Dermatofibroma	6.5	99.2	PRKCA, PRKCB, PRKCD, KIRREL, PDPN, CD63, LAMTOR1	not available
Osteosarcoma	4.6	65.2	p53, RB, BRCAness	palbociclib
Chondrosarcoma	4.6	81.9	IDH1/2, EXT1/2, EWS-NR4A3	sunitinib
Angiosarcoma	4.4	53.8	endoglin	carotuximab
Fibrosarcoma	3.9	82.9	COL1A1-PDGFB	imatinib, sunitinib
Stromal	3.9	75.6	JAZF1-SUZ12, YWHAE-NUTM2, ESR1	not available
Rhabdomyosarcoma	3.3	54.7	FGFR4, ALK1, PDGFRA, IGF1R, PAX3-FOXO1A	ponatinib, crizotinib, sorafenib, sunitinib, sphingosine
Other *	2.9	70.4	EWSR1-DDIT3, EWS-WT1, EWS-ATF1, MET, HGF, FOS	crizotinib, SU11274, AMG 102, FOS siRNA, ganitumab
Synovial	2.5	65.6	SS18, SS18-SSX1/2/4	tazemetostat
Malignant peripheral nerve sheath tumor	2.4	65.4	CSF1R kinase	PLX3397, sirolimus
Ewing sarcoma	2.3	64.0	IGF1R, FET-ETS	cituxumab

CSS: cause-specific survival; NOS: not otherwise specified; IGF1R: insulin-like growth factor 1 receptor; TK: tyrosine kinases; TKI: tyrosine kinase inhibitors; ALK: anaplastic lymphoma kinase; NTRK: neurotrophic receptor kinase; CDK4: Cyclin-dependent kinase 4; MDM2: mouse double minute 2 homolog; PDGFRA: platelet-derived growth factor receptor A; SQSTM1: sequestosome 1; VCL: vinculin; PRKCA/B/D: protein kinase C alpha/beta/delta; KIRREL: Kin Of IRRE Like; PDPN: podoplanin; LAMTOR1: late endosomal/lysosomal adaptor, MAPK and MTOR activator 1; RB: retinoblastoma; IDH1/2: isocitrate dehydrogenase 1/2; EXT1/2: exostosin-1/2; COL1A1: collagen type I alpha 1 chain; ESR1: estrogen receptor 1; HGF: hepatocyte growth factor; CSF1R: colony-stimulating factor 1 receptor; * including malignant mesenchymoma, odontogenic tumor, clear cell sarcoma, myxosarcoma, malignant hemangiopericytoma, malignant giant cell tumor, malignant granular cell tumor, alveolar soft part sarcoma, and desmoplastic small round cell tumor.

**Table 2:** Recruiting clinical trials combining immunotherapies with other treatment modalities (https://clinicaltrials.gov) * in adult patients (>18 years of age) with sarcoma.

Trial Registration Number	Title
Immunotherapy with radiation therapy	
NCT03307616	Nivolumab with and without ipilimumab and radiation therapy in treating patients with recurrent or resectable undifferentiated pleomorphic sarcoma or dedifferentiated liposarcoma before surgery
NCT03116529	Neoadjuvant durvalumab and tremelimumab plus radiation for high-risk STS (NEXIS)
NCT03463408	Immunotherapy (ipilimumab and nivolumab) and radiation in resectable STS
NCT03548428	Stereotactic body radiation therapy (SBRT) and atezolizumab versus SBRT (STEREOSARC)
**Immunotherapy with chemotherapy**	
NCT03899805	A phase II study of eribulin and pembrolizumab in STS
NCT03138161	SAINT: Trabectedin, ipilimumab and nivolumab as first line treatment for advanced STS
NCT03074318	Avelumab and trabectedin in treating patients with liposarcoma or leiomyosarcoma that is metastatic or cannot be removed by surgery
NCT03719430	APX005M and doxorubicin in advanced sarcoma
Immunotherapy with targeted therapy	
NCT04025931	A single-arm, open, phase II study of chidamide combined with toripalimab (anti-PD1) in refractory and advanced STS
NCT03190174	Nivolumab and ABI-009 (nab-rapamycin)

* As per 22nd of November 2020.

**Table 3:** Recruiting clinical trials with genetically engineered CAR T cells (https://clinicaltrials.gov) * in adult patients (>18 years of age) with sarcoma.

Trial Registration Number	Title
NCT03356782	Safety and efficacy evaluation of 4th generation safety-engineered CAR T-cells targeting sarcomas
NCT03960060	A Study of CCT301-59 CAR T therapy in adult subjects with recurrent or refractory solid tumors (including STS)
NCT03618381	EGFR806 CAR T cell immunotherapy for recurrent/refractory solid tumors in children and young adults
NCT03638206	Autologous NY-ESO-1 CAR-T cell immunotherapy for malignancies (including synovial sarcoma)
NCT03635632	C7R-GD2.CART cells for patients with relapsed or refractory neuroblastoma and other GD2 positive cancers (GAIL-N)
NCT00902044	HER2 Chimeric Antigen Receptor Expressing T Cells in Advanced Sarcoma

* As per 22nd of November 2020.

## Data Availability

No new data were created in this study.
